# Targeting Uremic Toxins in Chronic Kidney Disease: Current Challenges and Emerging Therapeutic Strategies

**DOI:** 10.3390/toxins18070311

**Published:** 2026-07-17

**Authors:** Regiane Stafim da Cunha, Paulo Cézar Gregório, Amanda Carina Coelho Morais, Fellype Carvalho Barreto, Andréa Emilia Marques Stinghen

**Affiliations:** 1 Laboratório de Nefrologia Experimental, Departamento de Patologia Básica, Universidade Federal do Paraná, Curitiba 81531-980, Brazil; regidacunha@ufpr.br (R.S.d.C.); paulocezargregorio@gmail.com (P.C.G.); 2 Escola de Ciências da Saúde, Centro Universitário Autônomo do Brasil, UniBrasil, Curitiba 82821-020, Brazil; 3 Hospital Universitário Regional de Maringá, Maringá 87083-240, Brazil; amandacoelho_med@hotmail.com; 4 Departamento de Clínica Médica, Programa de Pós-Graduação em Medicina Interna e Ciências da Saúde, Universidade Federal do Paraná, Curitiba 80060-900, Brazil; fellype.barreto@ufpr.br; 5 Serviço de Nefrologia, Complexo Hospital de Clínicas, Universidade Federal do Paraná, Curitiba 80060-900, Brazil

**Keywords:** uremic toxicity, cardiovascular disease, endothelial dysfunction, gut microbiota, dialysis therapies

## Abstract

Chronic kidney disease (CKD) represents a significant public health problem. Patients with CKD have high morbidity and mortality, mainly due to cardiovascular diseases. As CKD progresses, impaired renal clearance leads to the progressive accumulation of uremic toxins, culminating in deleterious effects on multiple organ systems. Uremic toxins contribute to cardiovascular disease by inducing inflammation, oxidative stress, and endothelial barrier dysfunction, thereby promoting atherosclerotic processes. They are also potentially implicated in neurological impairment, bone disorders, and sarcopenia. Some uremic toxins originate from dietary components and gut microbiota metabolism, which are key mechanisms and pathways in uremic toxicity. Current dialysis therapies only partially mitigate uremic toxicity, highlighting the need for strategies that reduce uremic toxin generation as well as more efficient dialysis modalities. Given the importance of uremic toxins in CKD progression, this review provides a comprehensive overview of uremic toxicity, focusing on its pathophysiological mechanisms, its relationship with the gut microbiota, and emerging interventions to mitigate its deleterious effects.

## 1. Introduction

Chronic kidney disease (CKD) has emerged as a major global public health challenge, affecting an estimated 850 million individuals worldwide and more than 10% of the adult population, while imposing a substantial burden in terms of morbidity, mortality, and healthcare expenditures [1,2]. Progressive deterioration of renal function disrupts fluid and electrolyte homeostasis and leads to the systemic accumulation of numerous metabolites that are normally cleared by the kidneys [3]. These retained compounds, collectively known as uremic toxins, progressively accumulate in the circulation and exert harmful biological effects on multiple organs and physiological systems [4,5]. It is noteworthy that the clinical burden of CKD extends far beyond the gradual decline in renal function itself. The heart–kidney axis is profoundly disrupted during CKD progression, as increased inflammation and oxidative stress contribute to the development of cardiorenal syndrome [6]. Importantly, patients with CKD face a markedly increased risk of cardiovascular disease, systemic inflammation, metabolic disturbances, and premature mortality, with cardiovascular complications representing the leading cause of death in this population [7,8].

CKD progression is strongly associated with renal fibrosis, defined by excessive extracellular matrix accumulation in the renal parenchyma, ultimately leading to progressive and irreversible loss of kidney function [9,10]. Transforming growth factor-β (TGF-β) is a key mediator of this profibrotic response, promoting the activation, proliferation, and differentiation of fibroblasts into myofibroblasts, thereby driving fibrogenesis [9,11]. Furthermore, the fibrotic process is profoundly influenced by inflammation and oxidative stress, both of which contribute to the progression of renal fibrosis [10]. In this context, macrophages play a central role in regulating inflammatory and profibrotic responses within the kidney [12,13]. As kidney function declines, the impaired clearance of uremic toxins leads to their progressive accumulation in the body. These compounds arise from endogenous metabolic processes as well as from dietary substrates that undergo transformation by the gut microbiota [14]. The retention of these solutes contributes to the development of the complex systemic condition known as uremia [4].

Over the past several decades, considerable efforts have been devoted to identifying and characterizing uremic retention solutes and to elucidating their biological effects. In this context, the European Uremic Toxin Work Group (EUTox) has played a pivotal role in cataloging these molecules and establishing a widely accepted classification system based on their physicochemical properties and dialytic removal characteristics [3,15]. According to this framework, uremic toxins can be broadly categorized into three major groups: small water-soluble compounds, middle molecules, and protein-bound uremic toxins (PBUTs) [3]. Among these categories, PBUTs have gained particular interest because of their strong association with adverse clinical outcomes and their limited removal by conventional dialysis techniques [4]. Notably, compounds such as indoxyl sulfate (IS) and *p*-cresyl sulfate (PCS) are largely derived from gut microbial metabolism of dietary amino acids and progressively accumulate as renal function declines [14,16]. Increasing evidence suggests that these molecules act not only as markers of impaired kidney function but also as active mediators of CKD-related complications. Indeed, uremic toxins have been implicated in the induction of oxidative stress, chronic inflammation, endothelial dysfunction, and vascular injury, processes that collectively contribute to the elevated cardiovascular risk observed in patients with CKD [4,17,18,19].

Experimental and clinical studies have demonstrated that exposure of endothelial cells to uremic toxins leads to structural and functional alterations of the vascular endothelium, including disruption of intercellular junctions, inflammatory activation, and increased permeability, all of which contribute to vascular damage and cardiovascular complications in CKD [18,19]. In addition, uremic toxins may promote the release of endothelial-derived extracellular vesicles that participate in intercellular communication and inflammatory signaling, further contributing to endothelial dysfunction and vascular injury [20]. Furthermore, metabolites generated through gut microbial metabolism, such as trimethylamine-N-oxide (TMAO), have also been associated with the development of atherosclerosis and cardiovascular events [21,22,23].

The clinical relevance of uremic toxins is further underscored by the limitations of current therapeutic approaches aimed at controlling toxin accumulation. Although conventional hemodialysis (HD) efficiently removes small water-soluble solutes, it is considerably less effective in eliminating middle molecules and PBUTs, largely because only the free (unbound) fraction of these compounds can cross the dialysis membrane [24]. As a result, many biologically active solutes continue to accumulate even in patients undergoing regular dialysis therapy, contributing to persistent systemic toxicity and sustained cardiovascular risk [4]. These limitations have stimulated increasing interest in alternative therapeutic strategies designed to reduce toxin generation, enhance toxin removal, or attenuate toxin-mediated biological effects. Approaches currently under investigation include adsorption-based therapies, modulation of gut microbiota to decrease toxin production, and pharmacological interventions targeting toxin-related signaling pathways [14,24].

Given the growing recognition of the central role of uremic toxins in the pathophysiology of CKD and its associated complications, a comprehensive understanding of their origin, classification, and biological activity is essential for the development of more effective therapeutic strategies. In this context, the present review provides an overview of current knowledge on uremic toxins in CKD, with particular emphasis on their classification, pathophysiological mechanisms, and emerging therapeutic approaches targeting toxin accumulation and toxicity. Furthermore, we discussed recent advances in the field, including microbiota-targeted interventions and precision medicine strategies that may contribute to improved management of patients with CKD. This review also serves as an introductory framework for the Special Issue “Treatment and Management of Uremic Toxins in Chronic Kidney Disease Patients”, highlighting emerging concepts and future therapeutic perspectives in this rapidly evolving field.

## 2. Classification of Uremic Toxins

Uremic toxins are solutes that accumulate in the body as kidney function declines, leading to harmful systemic effects. To be considered a uremic toxin, a compound must be reliably measurable in blood, plasma, or serum; exhibit elevated concentrations in patients with CKD compared to individuals without this disease; and demonstrate an association with clinically relevant CKD outcomes [5,25]. Regarding their origin, these compounds can be derived from endogenous metabolism, gut microbial activity, and dietary intake. However, the removal of the uremic toxins through dialysis therapies is still limited, highlighting the need for their precise characterization and a deeper understanding of their biological effects in CKD.

Uremic toxins are classically divided into three major groups: (I) small water-soluble molecules (<500 Da); (II) middle molecules (>500 Da); and (III) PBUTs (Table 1). This classification was first proposed by the European Uraemic Toxin Work Group (EUTox) in 2003, with the identification of 90 solutes [3,15]. As research has progressed over the years, the number of identified uremic toxins has expanded to approximately 150 [25]. Small water-soluble uremic toxins are efficiently cleared by conventional dialysis therapies, while middle-sized molecules require dialysis modalities with larger pore sizes. In contrast, PBUTs are poorly removed due to strong binding to serum proteins, mainly albumin.

Given advances in dialysis techniques over recent decades, Rosner et al. (2021) suggested a further stratification of middle-molecular-weight solutes into subcategories based on molecular weight [5]. The proposed revised classification is as follows: (I) small–middle molecules (0.5–15 kDa), including β2-microglobulin and parathyroid hormone (PTH); (II) medium–middle molecules (15–25 kDa), encompassing interleukin-6 (IL-6) and basic fibroblast growth factor (FGF-2); (III) large–middle molecules (25–58 kDa), represented by fibroblast growth factor 23 (FGF-23); and (IV) large molecules (58–170 kDa), such as modified albumin [5].

## 3. Pathophysiological Mechanisms

**Figure 1:**
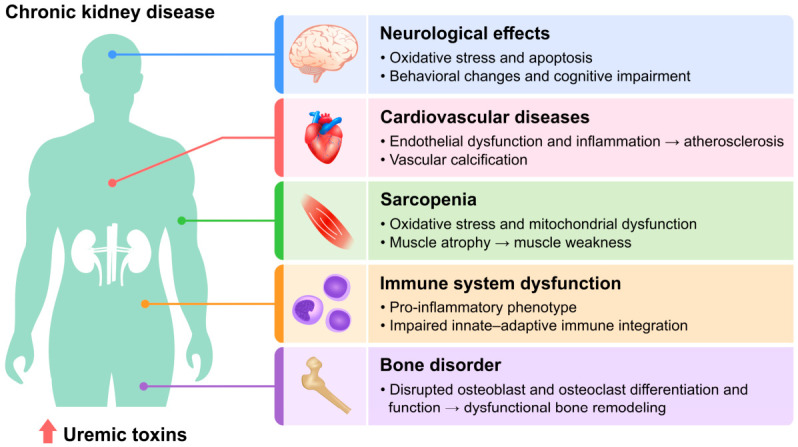
Pathophysiological effects of uremic toxins in chronic kidney disease (CKD). In CKD, reduced renal clearance leads to progressive accumulation of uremic toxins, which induce inflammation, oxidative stress, and cellular dysfunction, causing damage to various biological systems. Data from the literature has linked uremic toxins to neurological alterations, including behavioral changes and cognitive impairment. In the cardiovascular system, these toxins induce endothelial dysfunction, accelerate atherosclerosis, and promote vascular calcification, thereby increasing cardiovascular risk. Uremic toxin-induced oxidative stress and mitochondrial dysfunction also contribute to skeletal muscle wasting (sarcopenia). In addition, uremic toxins contribute to immune dysfunction by promoting a persistent pro-inflammatory phenotype and impaired integration of innate and adaptive immunity. They also impair bone homeostasis by altering osteoblast and osteoclast function, thereby contributing to the development of CKD-related mineral and bone disorders.

Uremic toxins cause negative effects on various organs and tissues, contributing to the outcomes of CKD progression. PBUTs, such as IS and PCS, have been extensively investigated in recent decades due to their effects on several cellular and molecular mechanisms and their difficulty of removal by conventional dialysis strategies [26]. Circulating PCS and IS levels were inversely correlated with eGFR, indicating an association with renal function decline in patients with CKD [16]. One of the most affected biological systems is the cardiovascular system, contributing to vascular dysfunction and cardiorenal syndrome [19,27]. Indeed, several clinical studies indicate that uremic toxins are associated with increased cardiovascular morbidity and mortality in patients with CKD, particularly IS, PCS, TMAO, and ADMA [22,28,29,30,31,32]. In vitro and in vivo evidence also indicates that uremic toxins could contribute to other comorbidities commonly observed in patients with CKD, including mineral and bone disorder, neurological impairment, immune system dysfunction, and sarcopenia (Figure 1).

### 3.1. Uremic Toxins Induce Endothelial Dysfunction

Endothelial dysfunction is a central mechanism in the pathogenesis of cardiovascular diseases, with strong evidence suggesting that uremic toxins contribute directly to this process in patients with CKD. Several studies have demonstrated that isolated uremic toxins or uremic serum trigger inflammatory responses and oxidative stress in endothelial cells, while also enhancing permeability.

Uremic toxins are known to induce the expression of pro-inflammatory molecules by endothelial cells, including monocyte chemoattractant protein-1 (MCP-1), E-selectin, vascular cell adhesion molecule 1 (VCAM-1), and intercellular adhesion molecule 1 (ICAM-1) [33,34,35,36]. In fact, these pro-inflammatory molecules are also elevated in patients with CKD, especially in the more advanced stages [37]. This increase is mainly driven by activation of the nuclear factor kappa B (NF-κB), a molecular pathway that has been shown to be stimulated by IS and TMAO [33,36,38]. The upregulation of these pro-inflammatory molecules results in enhanced leukocyte adhesion and migration, promoting vascular inflammation. In addition, damage to the endothelial glycocalyx is also associated with increased leukocyte adhesion and loss of vascular integrity [39]. Regarding this, higher levels of syndecan-1 and other markers of endothelial glycocalyx degradation are observed in patients with CKD and in animals treated with IS, indicating damage to the endothelium [40,41,42].

Elevated endothelial permeability is also observed in the context of uremic toxicity, an effect that is associated with disruption of intercellular junctions. One of the most important is the adherent junctions, composed mainly of VE-cadherin. Studies have demonstrated that PCS, IS, TMAO, and uremic serum modulate VE-cadherin, contributing to the loss of endothelial monolayer integrity [18,43,44,45].

In addition, uremic toxicity is closely linked to oxidative stress and reduced nitric oxide (NO) bioavailability, which plays a role in regulating vascular tone. In vitro studies have reported increased production of reactive oxygen species (ROS) in endothelial cells exposed to IS and PCS [38,46,47]. Furthermore, the uremic toxin ADMA inhibits the activation of endothelial nitric oxide synthase (eNOS), directly reducing NO production [48]. IS also reduces eNOS expression; however, it is proposed that this occurs via oxidative stress and the miRNA-92a pathway [49].

Cellular mechanisms that preserve endothelial function are also impaired by uremic toxins. In vitro studies have demonstrated that uremic serum as well as isolated PCS, IS, and advanced glycation end products (AGEs) suppress the Kruppel-like factor (KLF)-2 transcription factor, a key regulator of endothelial homeostasis [49,50,51]. KLF2 has anti-thrombotic, antioxidant, and anti-inflammatory effects, and its downregulation contributes directly to endothelial dysfunction and the development of atherosclerosis [52].

### 3.2. Extracellular Vesicles Formed in the Uremic Environment

Extracellular vesicles are nano- or micrometric structures enclosed by a plasma membrane that are released by cells under both physiological and pathological conditions. These vesicles play an important role in intercellular communication, carrying biomolecules from the cell of origin to another recipient cell. Studies have shown that the contents of extracellular vesicles are altered when cells are exposed to a uremic environment [53,54,55]. Most of these reported alterations are found in extracellular vesicles isolated from urine and blood, especially in their protein and miRNA content.

Characterization of alterations in extracellular vesicle composition is essential to elucidate their role in CKD pathogenesis and to identify potential biomarkers. Regarding this, patients with concomitant CKD and coronary artery disease exhibit reduced levels of the vasculoprotective miRNAs miR-130a-3p and miR-126-3p in circulating extracellular vesicles compared with patients presenting coronary artery disease alone [55]. In pediatric patients with CKD, circulating extracellular vesicles exhibit altered miRNA profiles, including reduced miR-126-3p levels [54]. Furthermore, endothelial extracellular vesicles formed in the presence of PCS and IS induce the expression of VCAM-1 on recipient endothelial cells, which may suggest a role in vascular dysfunction [20]. In vivo, Koide et al. (2023) found that circulating extracellular vesicles from an animal model of CKD induced calcification of aortic smooth muscle cells, with increased expression of osteogenic genes [56]. Analysis of their miRNA cargo also showed reduced levels of miR-16-5p, miR-17-5p, miR-20a-5p, and miR-106b-5p, which all have been associated with protective effects against vascular calcification [56]. Taken together, uremic toxins affect the content of extracellular vesicles, leading to changes in intercellular communication and downstream cellular responses.

**Figure 2:**
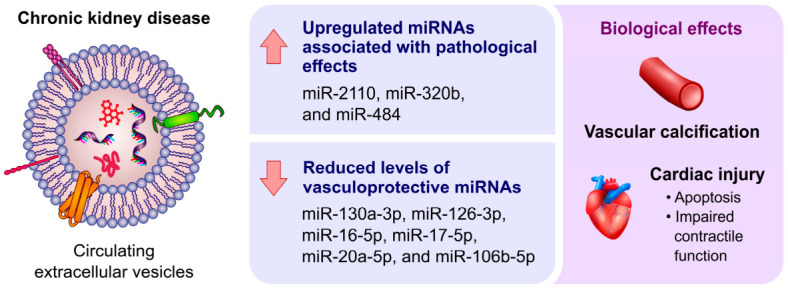
Altered microRNA cargo of circulating extracellular vesicles in chronic kidney disease (CKD). Uremic conditions modify the microRNA content of circulating extracellular vesicles, leading to reduced levels of vasculoprotective miRNAs and increased levels of miRNAs associated with pathological processes, such as inflammation, vascular calcification, and cardiac remodeling. Through intercellular communication mediated by extracellular vesicles, these altered miRNAs could contribute to cardiovascular complications by promoting vascular calcification and cardiac injury, including cardiomyocyte apoptosis and impairment of contractile function.

Recently, Li et al. (2026) demonstrated that circulating extracellular vesicles from plasma of patients with CKD induce cardiomyocyte injury in both in vitro and in vivo models, leading to increased apoptosis and impaired contractile function [53]. CKD is associated with altered circulating extracellular vesicle miRNA profiles, including increased miR-2110, miR-320b, and miR-484, which are associated with cardiac injury [53]. These findings support extracellular vesicles as mediators, biomarkers, and potential therapeutic targets in CKD. Figure 2 summarizes alterations in extracellular vesicle miRNA cargo and their association with pathological processes in CKD.

### 3.3. Bone Disorders

CKD progression is commonly accompanied by disturbances in mineral and bone metabolism, marked by dysregulation of calcium, phosphate, PTH, and vitamin D levels. These abnormalities directly affect bone health and contribute to cardiovascular calcifications [57]. In this context, several experimental studies suggest that uremic toxins may disrupt osteoblast and osteoclast differentiation and function [58,59]. Regarding this, IS and PCS have been shown to reduce cell viability and increase ROS production in osteoblasts in vitro [59,60,61,62]. In addition, IS reduced PTH receptor (PTHR) expression as well as suppressed PTH-induced intracellular cAMP generation in osteoblasts, which could be related to skeletal resistance to PTH [61]. Another uremic toxin, PCS, has also been shown to alter glutathione and glycerophospholipid pathways in osteoblasts, as reported by Wu et al. (2026) [63]. In vitro, osteoclast precursors exposed to IS exhibit suppressed RANKL-dependent differentiation into mature osteoclasts, which could contribute to abnormal bone turnover [58]. Taken together, these findings suggest that PBUTs, such as IS and PCS, could contribute to the pathogenesis of CKD-related bone disorders, at least in part, by inducing oxidative stress.

### 3.4. Neurological Effects Caused by Uremic Toxicity

Patients with CKD can develop a range of neurological complications, such as cognitive impairment, uremic encephalopathy, and seizures. Although the underlying mechanisms in CKD remain incompletely understood, experimental studies suggest that uremic toxins could contribute to central nervous system dysfunction, possibly through mechanisms involving oxidative stress [64,65]. In vitro, astrocytes exposed to IS exhibited increased ROS production, downregulation of cell-protective factors (including the NRF2 pathway), and enhanced apoptosis [66,67]. Furthermore, IS increased NF-kB activation and the expression of pro-inflammatory molecules [67]. IS, indole, IAA, and methylglyoxal reduced hippocampal neuronal cell viability and decreased glutathione levels, suggesting altered cellular redox balance [68]. In vivo, adenine-induced CKD rats also showed increased numbers of pyknotic hippocampal neurons and impaired learning process [68]. In another study conducted by Karbowska et al. (2020), rats exposed to IS in drinking water exhibited impairments in spatial memory and motor coordination, along with behavioral alterations such as apathetic behavior, increased stress sensitivity, and reduced locomotor and exploratory activity [69]. PCS also induced depression, anxiety, and cognitive impairment behaviors in nephrectomized mice [70].

### 3.5. Immune System Dysfunction

Uremic toxins disrupt the function of multiple cells of both the innate and adaptive immune systems, as demonstrated by experimental studies. In macrophages, IS induced the pro-inflammatory phenotype in vitro, suggesting that this uremic toxin acts towards the M1 macrophages [71,72]. Furthermore, PCS induced macrophage activation, but impaired antigen processing and weakened functional integration with the adaptive immune system [73]. Importantly, pro-inflammatory cytokines, such as tumor necrosis factor-alpha (TNF-α), released by IS-exposed monocytes could activate endothelial cells, thereby promoting vascular inflammation [74]. These data suggest impairment of cellular function, potentially contributing to both heightened inflammatory activity and increased susceptibility to infections.

### 3.6. Uremic Toxins Promote Sarcopenia

Patients with advanced CKD commonly develop sarcopenia. Studies have shown that uremic toxins may play a key role in this process by causing damage to skeletal muscle cells. One of the most studied uremic toxins is IS, which has been shown to be present at higher levels in muscle tissue in an animal model of CKD [75]. Experimental evidence has demonstrated that IS promotes metabolic alterations, such as glycolysis upregulation and mitochondrial dysfunction in myoblast cells [75]. Upon exposure to IS, these cells also show increased production of ROS, along with enhanced expression of pro-inflammatory mediators and genes associated with muscle atrophy [76]. Furthermore, uremic concentrations of IS induce apoptosis of myoblast cells [77].

Table 2 summarizes the biological effects of the main uremic toxins.

## 4. Gut Microbiota and Toxin Generation

The gut microbiota is currently recognized as an active metabolic organ with a central role in maintaining homeostasis and in the pathophysiology of several diseases, including CKD [14]. In this context, the gut–kidney axis represents a bidirectional interaction in which renal dysfunction alters the intestinal environment, promoting dysbiosis, while microbiota-derived metabolites contribute to the progression of kidney disease and its systemic complications [86,87]. This interaction is self-perpetuating: renal dysfunction disrupts the intestinal barrier and favors dysbiosis, as detailed below, while the resulting proteolytic environment increases the generation of gut-derived uremic toxins that further aggravate renal injury and fibrosis [88,89].

In CKD, significant alterations in microbial composition are observed, characterized by the expansion of proteolytic bacteria such as Enterobacteriaceae and Clostridiaceae, concomitant with a reduction in beneficial short-chain fatty acid (SCFA) producing microorganisms, including *Bifidobacterium* spp. and *Lactobacillus* spp. [90]. This shift compromises the production of essential metabolites, particularly butyrate, which serves as the primary energy source for colonocytes and plays a key role in maintaining intestinal barrier integrity and regulating local immune responses [91]. Consequently, dysbiosis redirects bacterial metabolism to proteolytic fermentation, favoring the generation of gut-derived uremic toxins that independently contribute to CKD progression and increased cardiovascular risk [92].

**Figure 3:**
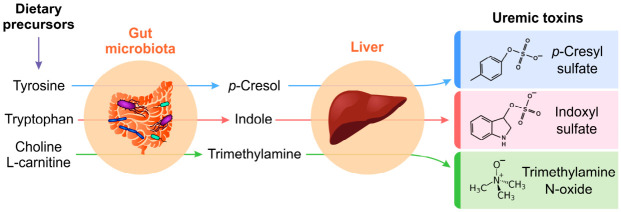
Formation of the uremic toxins *p*-cresyl sulfate (PCS), indoxyl sulfate (IS), and trimethylamine N-oxide (TMAO). These uremic toxins are diet-derived metabolites generated from precursors such as tryptophan, tyrosine, phenylalanine, choline, and L-carnitine. These compounds are metabolized by the gut microbiota and subsequently modified in the liver. Tyrosine is converted by intestinal bacteria into *p*-cresol, which is further metabolized in the liver into PCS, whereas tryptophan is converted into indole and subsequently conjugated into IS. Similarly, choline and L-carnitine are converted by gut microbiota into trimethylamine (TMA), which is oxidized in the liver to generate trimethylamine N-oxide (TMAO).

Microbial metabolism of aromatic amino acids in the colon represents the main pathway for the generation of PBUTs. Anaerobic bacteria expressing tryptophanase convert tryptophan into indole, which is absorbed and subsequently metabolized in the liver into IS and IAA [4,86]. Similarly, tyrosine and phenylalanine are converted into *p*-cresol, which is subsequently conjugated into PCS in the liver [86,92,93]. These toxins exert significant systemic effects, including the induction of oxidative stress and inflammation, thus contributing to renal injury progression and being associated with increased mortality in CKD patients [4,94,95]. In addition, IS and PCS have been shown to trigger the TGF-β1/Smad3 pathway, promoting the expression of Snail and inducing epithelial-to-mesenchymal transition (EMT) in tubular cells, which directly accelerates interstitial fibrosis [88,92]. Another relevant metabolic pathway involves the microbial conversion of choline and carnitine into trimethylamine (TMA), which is subsequently oxidized in the liver into TMAO [21,96]. Figure 3 summarizes the formation of these uremic toxins. Elevated TMAO levels are associated with increased cardiovascular risk and endothelial dysfunction, representing an important link between the gut microbiota and cardiovascular complications in CKD [43,96,97].

Beyond metabolic alterations, CKD also promotes structural disruption of the intestinal barrier. Urea accumulation in the bloodstream leads to increased diffusion into the intestinal lumen, where it is hydrolyzed by bacterial ureases into ammonia and ammonium hydroxide, resulting in elevated luminal pH and cytotoxic effects on tight junction proteins such as zonula occludens-1 (ZO-1) and occludins [92,98,99]. This process leads to epithelial barrier disruption, known as “leaky gut”, allowing the translocation of endotoxins such as lipopolysaccharide (LPS) into the systemic circulation [98,100]. Once in circulation, LPS activates inflammatory pathways mediated by NF-κB, promoting the release of pro-inflammatory cytokines such as IL-6 and TNF-α, as well as increased C-reactive protein levels, contributing to the chronic inflammatory state and elevated cardiovascular risk observed in CKD [90,94,101].

Diet represents the main exogenous modulator of gut microbiota composition and uremic toxin production. In patients with CKD, dietary restrictions on foods rich in potassium and phosphorus often lead to reduced fiber intake, thereby decreasing SCFA production and promoting proteolytic fermentation [14,91]. In this context, the dietary protein-to-fiber ratio emerges as an important determinant of circulating IS and PCS levels, more accurately reflecting toxin burden than isolated nutrient intake [102]. Clinical cohort data indicate that vegetarians exhibit significantly lower levels of IS and PCS than omnivores, largely due to higher fiber intake providing a substrate for saccharolytic bacteria to incorporate nitrogen into biomass rather than toxins [103,104]. Table 3 summarizes cross-sectional and interventional studies evaluating the effects of dietary protein, fiber, and plant-based diets on circulating PBUT levels. Dietary patterns such as the Mediterranean diet are inversely associated with PCS levels, while frequent vegetable consumption attenuates the impact of aromatic amino acid precursors on toxin production [104]. This effect is related to the stimulation of saccharolytic bacterial species such as *Faecalibacterium prausnitzii* and *Roseburias* spp., which favor nitrogen utilization for biomass synthesis rather than toxic metabolite production [90,105]. Therefore, increased fiber intake contributes to restoring intestinal metabolic balance and reducing uremic toxicity [87,104].

Evidence also indicates that gut microbiota composition independently influences symptom burden in CKD. Specific bacterial genera, such as *Acetanaerobacterium* spp. and *Clostridia innocuum*, have been associated with greater symptom severity, including fatigue and pruritus, suggesting that the microbiome modulates the clinical phenotype of uremia through mechanisms that are not yet fully understood [87].

The recognition of the gut–kidney axis has driven the development of therapeutic strategies targeting the microbiota, including dietary interventions, prebiotics, probiotics, and fecal microbiota transplantation [109]. However, the clinical translation of prebiotics and probiotics remains challenging: clinical trials in CKD have produced conflicting results even across meta-analyses, with some reporting significant reductions in circulating PCS but not IS, while a randomized, double-blind, placebo-controlled crossover trial of arabinoxylan oligosaccharides found no significant effect on either toxin [110,111]. Notably, the only long-term (12-month) randomized controlled trial of synbiotic supplementation in non-dialysis CKD patients found no significant reduction in uremic toxins but instead observed a greater decline in eGFR and rise in serum creatinine compared with placebo, raising a safety signal that warrants further investigation [112]. Among these approaches, Lactobacillus casei Zhang has demonstrated nephroprotective properties by modulating the gut microbiome, increasing SCFA and nicotinamide availability, reducing the expression of pro-fibrotic genes and the profibrogenic cytokine TGF-β1, and attenuating renal inflammation [113,114].

Collectively, these findings highlight that modulation of the gut microbiota through diet and targeted interventions represents a promising strategy to reduce systemic inflammation, decrease uremic toxin production, and slow CKD progression and its associated cardiovascular complications [90,95,115].

## 5. Therapeutic Strategies

The main strategies commonly used to reduce the concentration of uremic solutes in patients with CKD stage 5D are conventional HD and peritoneal dialysis (PD), methods considered to have low efficiency for the removal of middle-molecular-weight and PBUTs [24]. PD, due to its transport characteristics across the peritoneal membrane, appears to provide fewer pores with a larger radius compared with the artificial membrane of conventional HD. This feature favors greater clearance of middle-sized molecules, in addition to better preserving residual renal function, a relevant determinant of uremic solute clearance [116,117]. However, neither HD nor PD efficiently removes highly PBUTs such as IS and PCS, whose strong albumin binding limits diffusive clearance severely [118,119].

Dialytic therapies, such as hemodiafiltration (HDF), which combine solute removal by convection and diffusion, have been investigated with the aim of improving the removal of these toxins. Although higher convective volumes in HDF consistently increase the clearance of β2-microglobulin and other middle molecules [120,121], it is important to note that randomized clinical trials have reported inconsistent effects on mortality and cardiovascular outcomes compared with the use of high-flux membranes [122,123], except in selected patient subgroups [124,125]. Similarly, inconsistent findings have been reported for other uremic solutes, including leptin and AGEs [126]. Therefore, improved biochemical clearance should not be interpreted as synonymous with improved clinical outcomes.

The development of dialytic membranes with medium cut-off (MCO) or high cut-off (HCO), with pore sizes of approximately 5 nm and 10 nm, respectively, has enabled more efficient removal of large middle molecules [127,128]. MCO membranes optimize the clearance of solutes up to approximately 45 kDa while minimizing clinically relevant albumin loss, giving rise to the technique known as expanded hemodialysis (HDx) [129]. HCO membranes, although highly effective in removing large molecules, are associated with significant albumin loss, limiting their routine use to specific scenarios [129]. Randomized clinical studies have consistently demonstrated superior removal of middle molecules with MCO membranes compared with conventional high-flux HD, with overall performance approaching that of online HDF [127,130]. However, improvements in PBUT clearance remain modest, and evidence demonstrating superior long-term clinical outcomes with MCO membranes is still lacking. Accordingly, the advantages and limitations of currently available extracorporeal therapies are summarized in Table 4.

Kidney transplantation remains the most effective renal replacement therapy for reducing or normalizing uremic toxin concentrations [131,132]. Beyond restoring renal clearance, recovery of kidney function is thought to contribute to the partial re-establishment of the gut–kidney axis by reducing intestinal exposure to urea, improving epithelial barrier integrity, and promoting recovery of gut microbial diversity, thereby decreasing the generation of gut-derived uremic toxins, particularly PCS and IS [131]. However, restoration of the intestinal microbiota is often incomplete, as immunosuppressive therapy, antibiotic exposure, dietary modifications, and persistent metabolic disturbances continue to shape microbial composition after transplantation [133]. Although kidney transplantation reduces circulating concentrations of PBUTs, relatively few studies have evaluated their association with clinically relevant outcomes, such as allograft function, cardiovascular events, and patient survival [132]. Consequently, the prognostic significance of persistent uremic toxins in kidney transplant recipients remains incompletely understood and represents an important area for future investigation.

The concept that reducing the generation of uremic toxins is as relevant as their removal has gained prominence, leading to the expansion of research from a purely “clearance-based” model to an integrated model that includes the intestine as a therapeutic target [24]. Therapies targeting the intestinal microbiota, aiming to reduce the burden of gut-derived uremic toxins, involve three axes: diet, microbiota modulation, and reduction in absorption. The implementation of a low animal-protein diet supplemented with ketoacids and a vegetarian diet has been associated with lower serum levels of IS and PCS, respectively [103,108]. Reductions in circulating concentrations of indoles and phenols following the use of prebiotics, probiotics, and synbiotics have been reported in some studies, likely by increasing the saccharolytic activity of colonic bacteria and reducing the production of proteolytic fermentation metabolites [134,135]. However, the evidence remains heterogeneous due to differences in bacterial strains, formulations, treatment duration, dietary interventions, and CKD stage. Well-designed randomized clinical trials are still needed before these therapies can be routinely recommended for the reduction of uremic toxins.

AST-120, a carbon-based oral adsorbent, acts by binding toxin precursors in the intestinal lumen, reducing their systemic absorption. Experimental studies have shown that AST-120 may modulate the intestinal microbiota, reducing *p*-cresol-producing bacteria, which may partly explain its greater effect on PCS concentrations [136]. Despite these promising biological mechanisms, the two largest randomized placebo-controlled trials evaluating AST-120 (EPPIC-1 and EPPIC-2), involving more than 2000 patients with CKD, failed to demonstrate significant benefit for their primary composite renal endpoints. Subsequent post hoc analysis has shown a protective effect on renal function in the subgroup of patients with hematuria receiving ACE inhibitors/ARBs; however, these findings should be regarded as hypothesis-generating rather than confirmatory [137]. More recently, meta-analyses have suggested modest reductions in composite renal outcomes and progression to kidney failure, particularly in Asian populations [138]. Nevertheless, these pooled estimates should be interpreted cautiously and therefore do not outweigh the negative findings of the pivotal randomized trials. Consequently, although AST-120 remains a biologically attractive strategy for reducing gut-derived uremic toxins, current evidence is insufficient to support its routine use worldwide.

Sevelamer, an agent traditionally used for the control of hyperphosphatemia, seems to have pleiotropic effects, leading to lower systemic inflammation and oxidative stress, in comparison with calcium salts [139]. In vitro studies have demonstrated adsorption of AGEs, indole, *p*-cresol, IS, and IAA [35,140,141]. However, these experimental findings have not been consistently reproduced in vivo, and reductions in circulating PBUT concentrations have been variable across clinical studies [139,141]. Importantly, the beneficial effects reported with sevelamer may also result from mechanisms unrelated to direct toxin adsorption, including improved phosphate control, reduced vascular calcification and attenuation of systemic inflammation [35,139]. Therefore, although sevelamer represents a promising adjunctive therapy, current evidence remains insufficient to conclude that it consistently reduces circulating PBUT levels in patients with CKD.

## 6. Future Perspectives

Adsorption-based technologies represent a promising complementary strategy, particularly for PBUT removal, since conventional dialysis eliminates only their free circulating fraction. However, current evidence is largely restricted to in vitro studies and early translational investigations, and several important barriers remain before routine clinical implementation [142,143]. Competitive displacement of PBUTs from albumin has emerged as another promising strategy. In silico models have demonstrated greater efficacy of these therapies compared with HDF or sorbents in the dialysate [144]. Early clinical studies have shown a significant increase in the clearance of IS and PCS following the infusion of ibuprofen, as an albumin-binding competitor, in the arterial line during HD [145]. Nevertheless, concerns regarding drug toxicity, pharmacokinetic variability, and potential adverse effects currently preclude clinical application.

Likewise, free fatty acids such as oleic acid, linoleic acid, and octanoate have demonstrated increased PBUT removal in experimental dialysis systems [146,147]. However, these findings remain confined to preclinical models, and issues related to metabolic toxicity and membrane instability must be resolved before clinical translation. Therefore, these approaches should currently be regarded as promising investigational strategies rather than therapies ready for routine clinical practice.

Advances in multi-omics technologies, particularly metabolomics, have the potential to enable individualized characterization of the uremic phenotype through the identification of specific metabolic signatures. In the future, this approach may support precision medicine strategies by guiding the selection of dietary interventions, microbiota-modulating therapies, intestinal adsorbents, and extracorporeal treatment modalities according to the predominant metabolic characteristics of each patient [148,149]. However, significant challenges must still be overcome before these approaches can be incorporated into routine clinical practice.

In addition to the development of novel extracorporeal and adsorption technologies, the future management of uremic toxicity will likely require an integrated strategy that combines more efficient toxin removal with interventions aimed at reducing the generation of these compounds, particularly as knowledge of the gut–kidney axis continues to expand. In this context, a deeper understanding of the complex interactions among the broad spectrum of uremic toxins will be essential to translate mechanistic discoveries into effective personalized therapies capable of improving cardiovascular outcomes, slowing the progression of CKD, and enhancing patients’ quality of life.

## 7. Conclusions

Uremic toxins are key mediators of CKD progression and its associated complications. Growing evidence indicates that these compounds are active participants in the pathophysiological processes that drive adverse clinical outcomes. Experimental data has shown that uremic toxins contribute to cardiovascular dysfunction, inflammation, oxidative stress, immune dysregulation, neurological impairment, bone disorders, and sarcopenia. Advances in the understanding of uremic toxicity have highlighted the central role of the gut–kidney axis and reinforced the need for therapeutic strategies that extend beyond toxin removal alone. Although current dialysis modalities provide partial control of toxin accumulation, emerging approaches targeting gut microbiota modulation, intestinal toxin generation, adsorption technologies, and personalized interventions offer promising perspectives. Continued integration of mechanistic, clinical, and omics-based research will be essential to improve patient stratification, identify novel therapeutic targets, and ultimately reduce the burden of uremic toxicity in patients with CKD.

## Figures and Tables

**Table 1:** Classification of uremic toxins.

Group	Main Prototypes
Small water-soluble molecules	Urea, uric acid, creatinine, trimethylamine-N-oxide (TMAO), asymmetric dimethylarginine (ADMA), guanidine, guanidinosuccinic acid
Middle molecules	β2-microglobulin, parathyroid hormone (PTH), leptin, adiponectin, basic fibroblast growth factor (FGF-2), cystatin C, hyaluronic acid, endothelin
Protein-bound molecules	Indoxyl sulfate (IS), indole-3-acetic acid (IAA), p-cresyl sulfate (PCS), p-cresyl glucuronide (PCG), hippuric acid (HA), kynurenic acid (KA), 3-carboxy-4-methyl-5-propyl-2-furanpropionic acid (CMPF), homocysteine, methylglyoxal

**Table 2:** Overview of the biological effects of major uremic toxins.

Uremic Toxin	Source	Removal by Dialytic Therapies	Main Biological Effects
Trimethylamine-N-oxide (TMAO)	Choline and carnitine metabolism by gut microbiota [78,79]	Moderate	Atherosclerosis, cardiovascular risk [21,22]
Asymmetric dimethylarginine (ADMA)	Endogenous metabolism	Moderate	Reduced NO bioavailability, endothelial dysfunction [48]
β2-microglobulin	Endogenous protein turnover	Moderate	Dialysis-related amyloidosis, inflammation [80]
Indoxyl sulfate (IS)	Tryptophan metabolism by gut microbiota [81]	Very low	Oxidative stress, endothelial dysfunction [33,38]
*p*-Cresyl sulfate (PCS)	Tyrosine and phenylalanine metabolism by gut microbiota [82]	Very low	Inflammation, vascular injury, atherosclerosis [83,84,85]

**Table 3:** Protein-bound uremic toxin levels according to dietary patterns in chronic kidney disease

Dietary Comparison	Study Design	Main Findings on IS and PCS	Reference
Vegetarian vs. omnivore diet	Cross-sectional study	Vegetarians exhibited significantly lower circulating free PCS and IS levels compared with omnivores.	Patel et al. (2012) [103]
Dietary protein-to-fiber ratio	Cross-sectional study, n = 40 (CKD)	A higher protein-to-fiber ratio was independently associated with higher serum IS and PCS, beyond the effect of protein or fiber intake alone.	Rossi et al. (2015) [102]
Dietary intake of tyrosine and phenylalanine (protein precursors)	Cross-sectional study, n = 27 (non-dialysis CKD)	Tyrosine and phenylalanine intake positively correlated with plasma PCS (r = 0.58 and r = 0.53, respectively; *p* < 0.01), independent of eGFR and age.	Fernandes et al. (2020) [106]
Dietary fiber intake and plant-based diet quality	Cross-sectional study, n = 68 (CKD)	Higher dietary fiber intake was associated with lower total IS; a healthier plant-based diet index was associated with lower free PCS.	McFarlane et al. (2022) [107]
Very low-protein diet (VLPD) vs. free diet	Interventional study	VLPD significantly reduced serum IS levels compared with a free diet.	Marzocco et al. (2013) [108]

IS, indoxyl sulfate; PCS, *p*-cresyl sulfate; CKD, chronic kidney disease; eGFR, estimated glomerular filtration rate; VLPD, very low-protein diet.

**Table 4:** Comparison of extracorporeal therapies for uremic toxin removal.

Therapy	Small Water-Soluble Toxins	Middle Molecules	Protein-Bound Uremic Toxins	Main Advantages	Main Limitations	Typical Clinical Indications
Conventional HD	Excellent	Limited	Poor	Widely available; efficient small-solute removal	Limited PBUT and middle-molecule clearance	Standard chronic dialysis
Peritoneal dialysis	Good	Moderate	Poor	Better preservation of residual kidney function	Limited PBUT removal; lower efficiency for large molecules	Home dialysis; patients with preserved residual kidney function
High-flux HD	Excellent	Better than low-flux HD	Poor	Improved β2-microglobulin removal	No consistent survival benefit demonstrated	Standard chronic dialysis
Online HDF	Excellent	Excellent	Limited	Superior middle-molecule clearance through convection	Requires high blood flow and ultrapure water; inconsistent RCT results for mortality	Selected chronic HD patients
Expanded HD (MCO membrane)	Excellent	Excellent	Limited	Improved clearance of large middle molecules without significant albumin loss	Limited PBUT removal; long-term clinical benefit remains uncertain	Alternative when HDF is unavailable
High cut-off membrane	Excellent	Excellent	Limited	Very high permeability for large molecules	Clinically relevant albumin loss	Acute kidney injury, myeloma cast nephropathy, selected inflammatory conditions

HD, hemodialysis; HDF, hemodiafiltration; MCO, medium cut-off; PBUT, protein-bound uremic toxin; RCT, randomized controlled trial.

## Data Availability

No new data were created or analyzed in this study. Data sharing is not applicable to this article.

## Abbreviations

The following abbreviations are used in this manuscript:

ADMA	Asymmetric dimethylarginine
AGEs	Advanced glycation end products
CKD	Chronic kidney disease
CMPF	3-carboxy-4-methyl-5-propyl-2-furanpropionic acid
EMT	Epithelial-to-mesenchymal transition
eNOS	Endothelial nitric oxide synthase
FFAs	Free fatty acids
FGF	Fibroblast growth factor
HA	Hippuric acid
HD	Hemodialysis
HDF	Hemodiafiltration
HMW	High molecular weight
IAA	Indole-3-acetic acid
ICAM-1	Intercellular adhesion molecule 1
IL-6	Interleukin-6
IS	Indoxyl sulfate
KA	Kynurenic acid
KLF	Kruppel-like factor
LPS	Lipopolysaccharide
MCO	Medium cut-off
MCP-1	Monocyte chemoattractant protein-1
NO	Nitric oxide
NF-κB	Nuclear factor kappa B
PBUTs	Protein-bound uremic toxins
PCG	P-cresyl glucuronide
PCS	P-cresyl sulfate
PD	Peritoneal dialysis
PTH	Parathyroid hormone
RCT	Randomized controlled trials
ROS	Reactive oxygen species
SCFA	Short-chain fatty acid
TGF-β	Transforming growth factor-β
TMA	Trimethylamine
TMAO	Trimethylamine-N-oxide
TNF-α	Tumor necrosis factor-alpha
VCAM-1	Vascular cell adhesion molecule 1
VLPD	Very low-protein diet
ZO-1	Zonula occludens-1
